# Association of *CCR2-CCR5* Haplotypes and *CCL3L1* Copy Number with Kawasaki Disease, Coronary Artery Lesions, and IVIG Responses in Japanese Children

**DOI:** 10.1371/journal.pone.0011458

**Published:** 2010-07-07

**Authors:** Manju Mamtani, Tomoyo Matsubara, Chisato Shimizu, Susumu Furukawa, Teiji Akagi, Yoshihiro Onouchi, Akira Hata, Akihiro Fujino, Weijing He, Sunil K. Ahuja, Jane C. Burns

**Affiliations:** 1 South Texas Veterans Health Care System and Department of Medicine, The Veterans Administration Center for AIDS and HIV-1 Infection, University of Texas Health Science Center at San Antonio, San Antonio, Texas, United States of America; 2 Department of Pediatrics, Juntendo University Graduate School of Medicine, Chiba, Japan; 3 Department of Pediatrics, Rady Children's Hospital, University of California San Diego School of Medicine, La Jolla, California, United States of America; 4 Department of Pediatrics, Yamaguchi University Graduate School of Medicine, Yamaguchi, Japan; 5 Pediatrics Cardiac Care Unit, Okayama University Hospital, Okayama, Japan; 6 Laboratory for Cardiovascular Diseases, Center for Genomic Medicine, RIKEN, Kanagawa, Japan; 7 Department of Public Health, Chiba University Graduate School of Medicine, Chiba, Japan; 8 Department of Surgery, National Center for Child Health and Development, Tokyo, Japan; 9 Departments of Microbiology and Immunology, and Biochemistry, University of Texas Health Science Center, San Antonio, Texas, United States of America; Innsbruck Medical University, Austria

## Abstract

**Background:**

The etiology of Kawasaki Disease (KD) is enigmatic, although an infectious cause is suspected. Polymorphisms in CC chemokine receptor 5 (CCR5) and/or its potent ligand CCL3L1 influence KD susceptibility in US, European and Korean populations. However, the influence of these variations on KD susceptibility, coronary artery lesions (CAL) and response to intravenous immunoglobulin (IVIG) in Japanese children, who have the highest incidence of KD, is unknown.

**Methodology/Principal Findings:**

We used unconditional logistic regression analyses to determine the associations of the copy number of the *CCL3L1* gene-containing duplication and *CCR2-CCR5* haplotypes in 133 Japanese KD cases [33 with CAL and 25 with resistance to IVIG] and 312 Japanese controls without a history of KD. We observed that the deviation from the population average of four *CCL3L1* copies (i.e., < or > four copies) was associated with an increased risk of KD and IVIG resistance (adjusted odds ratio (OR)  = 2.25, p = 0.004 and OR = 6.26, p = 0.089, respectively). Heterozygosity for the *CCR5* HHF*2 haplotype was associated with a reduced risk of both IVIG resistance (OR = 0.21, p = 0.026) and CAL development (OR = 0.44, p = 0.071).

**Conclusions/Significance:**

The *CCL3L1-CCR5* axis may play an important role in KD pathogenesis. In addition to clinical and laboratory parameters, genetic markers may also predict risk of CAL and resistance to IVIG.

## Introduction

Kawasaki disease (KD) is an acute, self-limiting systemic vasculitis of infants and children [1], [2]. The most serious complication of KD is the development of coronary artery lesions (CAL) that range from transient dilatation to destruction of the vessel wall architecture resulting in aneurysms [3]. Indeed, the primary goal of KD treatment is to prevent this complication [1], [2]. There is significant inter-individual variation in KD susceptibility as well as CAL development. Moreover, although administration of a combination of a high dose intravenous immunoglobulin (IVIG) and aspirin is the standard therapy for acute KD, 15–30% of KD patients have persistent or recurrent fever after IVIG treatment [4], [5], [6], [7], [8], [9], [10]. Also, such patients are at increased risk of developing CAL [11]. Thus, identification of host factors that influence KD susceptibility, CAL development and resistance to IVIG treatment may provide new insights into KD pathogenesis, novel means for prognostication of clinical outcome, and therapeutic targets.

According to a current paradigm, KD is thought to be triggered by an infectious agent that elicits an inflammatory response directed at cardiovascular tissues in genetically susceptible hosts [1], [12], [13]. Polymorphisms in various genes have been shown to influence KD susceptibility in different populations [14], [15], [16], [17], [18], [19], [20], [21], [22]. Similarly, variations in the genes encoding CD14 [23], matrix metalloproteinase (MMP)-3 [24], vascular endothelial growth factor (VEGF) and its receptor kinase insert domain receptor (KDR) [21] have been implicated in CAL development in KD. With respect to response to IVIG, several studies have reported laboratory and demographic predictors associated with IVIG failure [6], [7], [8]. However, the generalization of scoring systems based on such predictors to multiethnic U.S. populations has not been successful [10]. The genetic basis of IVIG resistance in the setting of KD or other inflammatory, autoimmune and infectious diseases in which IVIG has been empirically used (e.g. Idiopathic thrombocytopenic purpura), including pediatric HIV and post-infectious complications [25], has not been fully elucidated.

There is evidence to suggest that recruitment of inflammatory cells in KD may be mediated through CC chemokine receptor 5 (CCR5) [15], [19], [26]. Chemotactic gradients for homing of CCR5+ cells are provided by a variety of chemokines, the most potent of which is its ligand - CC ligand 3 like 1 (CCL3L1) [27]. The genes encoding CCR5 and CCL3L1 demonstrate two distinct types of polymorphisms: single nucleotide polymorphisms in *CCR5* [28] and copy number variation (CNV) in the *CCL3L1-*gene containing segmental duplication [29]. There is a growing interest in understanding the contribution of CNV in disease pathogenesis since it is recognized that 12% of the human genome may have undergone segmental duplications [30], [31]. We previously found that variations in *CCR5* and *CCL3L1* affect susceptibility to KD in parent-child trios from the United States [15].

However, there is significant variation in the prevalence of KD as well as the frequency of *CCR5* genotypes and *CCL3L1* copy number in different populations [15], [27], [32]. Consequently, whether the observations made in US trios can be generalized to Japanese children is unknown. To address this, we conducted a case-control study in subjects from Japan, a geographic region where the prevalence of KD is at least 10 times higher than the Western world [1], [2]. We tested the hypothesis that *CCR5* haplotypes and *CCL3L1* copy number influence KD susceptibility and two disease-related outcomes: development of CAL and IVIG resistance.

## Materials and Methods

### Ethics Statement

This study was approved by the institutional review boards of Yamaguchi and Kurume University Hospitals in Japan and the University of California San Diego and the University of Texas Health Science Center in San Antonio in the U.S. and written informed consent was given by the parents of all KD subjects and controls.

### Study subjects

We conducted an unmatched case-control study of 133 cases of KD and 312 controls collected between January 2002 and April 2005. The KD patients were recruited from three sites: the Department of Pediatrics, Yamaguchi University Hospital; Oita Children's Hospital; and Kurume University Hospitals, Japan. All patients met the Japanese criteria for the diagnosis of KD [33]. CAL was defined as a luminal diameter >3 mm for patients <4 years or >4 mm for patients >5 years of age, or an internal diameter of one or more segments at least 1.5 times larger than the adjacent segment [34]. IVIG-resistant subjects were defined as KD patients who had persistent fever (≥38.0°C) for at least 36 hours after completion of the IVIG infusion and who received secondary treatment after the initial treatment with IVIG. KD patients who did not receive secondary treatment were considered to have responded to the initial IVIG treatment. The initial IVIG was administered as a single infusion of 2 g/kg/day. All KD patients also received oral aspirin (30 mg/kg/d). Controls were Japanese adults without a history of KD recruited from three centers: San Diego, CA, and Yamaguchi University and RIKEN in Tokyo, Japan. Most of the controls of Japanese origin (28% from Yamaguchi University, 60% from Riken, and 12% from San Diego) were healthy adults and some had common diseases of adulthood unrelated to KD.

### Genotyping

The methods for genotyping *CCR5* polymorphisms are described elsewhere [15], [27], [32]. The variations in *CCR5* were categorized into haplotypes as described previously and were designated as *CCR5* human haplogroups A (HHA), HHB, HHC, HHD, HHE, HHF*1, HHF*2, HHG*1, and HHG*2 [35]. The *CCR5* haplotypes that bear the *CCR5*-Δ32 or *CCR2*-64I polymorphisms are designated as the *CCR5* HHG*2 and HHF*2 haplotypes, respectively [32], [35] Copy number of the *CCL3L1* gene-containing segmental duplication was estimated as described previously [27]. The assay used to genotype *CCL3L1* copy number captures three separate *CCL3L* genes (*CCL3L1*, *CCL3L2* and *CCL3L3*) as described previously [27].

### Statistical analysis

Allele frequency and Hardy-Weinberg equilibrium for all the *CCR5* haplotypes was estimated using the PowerMarker software [36]. We used unconditional multiple logistic regression analysis to evaluate the association of *CCR5* haplotypes and *CCL3L1* copy number with KD-related outcomes. The median number of *CCL3L1* copies in the study population was 4 and for this reason the study subjects were trichotomized into those possessing <4, 4 and >4 *CCL3L1* copies. In these regression analyses, we included *CCR5* haplotypes (HHA, HHC, HHE, HHF*1, HHF*2 and HHG1) and *CCL3L1* copy number (less than 4 and greater than 4) in the same regression model. To determine whether *CCL3L1* gene copy number modified the KD-influencing effects of *CCR5* haplotypes, we used the Mantel-Haenszel test of homogeneity. We used Stata 10.0 (Stata Corp, College Station, Texas) for the statistical analysis.

## Results

Among the cases there were 55 (41.35%) females and 78 (58.65%) males whereas in the control group there were 190 (60.90%) females and 122 (39.10%) males. KD patients with available echocardiographic data were categorized into 2 groups according to the presence of CAL. There were 33 (27.5%) and 87 (72.5%) patients with and without CAL, respectively. Mean age of disease onset was 43.5 months (range 2–270 months). Of the 95 cases who were treated with IVIG within the first 10 days of onset of fever, 25 (26.32%) were resistant to treatment.

**Figure 1:**
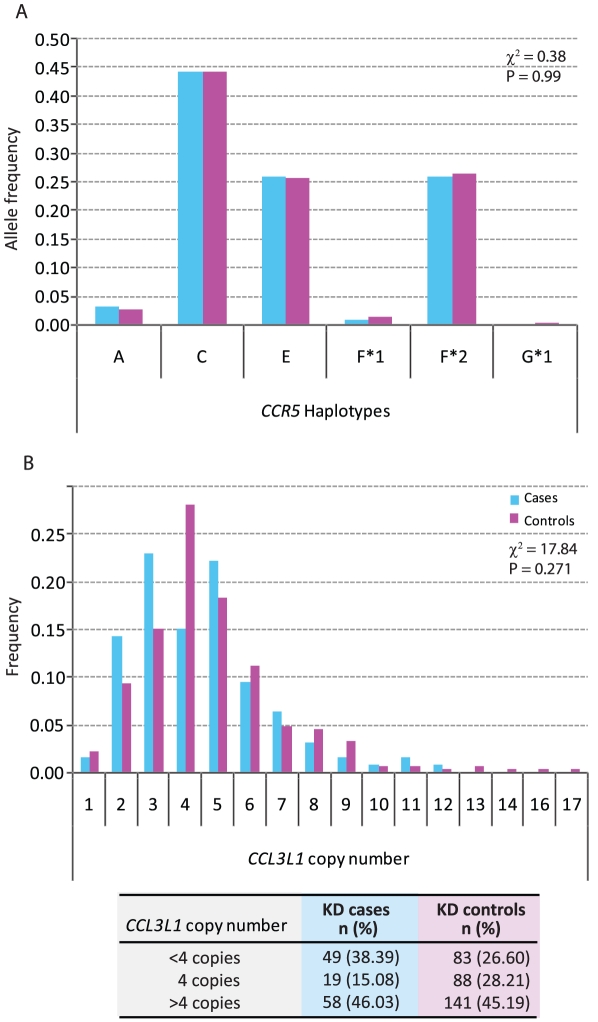
Distribution of *CCR5* haplotypes and *CCL3L1* copy number in cases (blue bars) and controls (purple bars). (A) Distribution of *CCR5* haplotypes and (B) Distribution of *CCL3L1* copy number. The overall difference of distribution between cases and controls was tested for significance using the χ^2^ test. The table at the bottom of Panel B shows frequencies of *CCL3L1* copy number categories in cases and controls. The categories were derived since 4 was the median copy number in the study population.

The most common *CCR5* haplotype was *CCR5* HHC, followed by HHF*2 and HHE (Fig. 1A). In the Japanese population the HHG*2 haplotype bearing the *CCR5*-Δ32 mutation is very rare. The *CCR5* locus was in Hardy-Weinberg equilibrium (Exact P = 0.9808 in controls and 0.5624 in cases). The median *CCL3L1* copy number in both cases and controls was four (Fig. 1B).

To determine whether *CCR5* haplotypes or copy number of the *CCL3L1* gene-containing segemental duplication was associated with an altered risk of developing KD, we first performed stepwise unconditional logistic regression analyses. We found that both possession of <4 (OR = 2.73, 95% CI = 1.49–5.03, p = 0.001) and >4 *CCL3L1* copies (OR = 1.91, 95% CI = 1.06–3.41, p = 0.03) was associated with an increased risk of developing KD (Table 1, Final model). Since gender distribution in the case and control groups was different, we adjusted for this covariate, and the adjusted odds ratios indicated that possession of <4 (OR_adjusted_ = 2.64, 95% CI = 1.42–4.88, p = 0.002) and >4 *CCL3L1* copies (OR_adjusted_ = 2.00, 95% CI = 1.11–3.61, p = 0.022) remained associated with a significantly higher risk of developing KD. Thus, departure from the population average of 4 *CCL3L1* copies (i.e., either < or >4 copies) was associated with a significantly increased risk of KD before (OR = 2.21, 95% CI = 1.28–3.82, p = 0.004) and after adjustment for gender (OR_adjusted_ = 2.25, 95% CI = 1.29–3.91, p = 0.004).

**Table 1:** Association of *CCR5* haplotypes and *CCL3L1* copy number with Kawasaki disease susceptibility.

*CCR5* haplotype/*CCL3L1* copy number	OR	95% CI	P value
Full Model			
*CCR5* HHA	1.13	0.44–2.87	0.802
*CCR5* HHC	0.70	0.39–1.26	0.236
*CCR5* HHE	0.80	0.47–1.36	0.407
*CCR5* HHF*1	0.58	0.12–2.91	0.512
*CCR5* HHF*2	0.75	0.44–1.27	0.283
*CCL3L1* <4copies	2.71	1.47–4.99	0.001
*CCL3L1* >4 copies	1.90	1.06–3.42	0.031
Final Model (Probability Criterion of 0.1)			
*CCL3L1* <4copies	2.73	1.49–5.03	0.001
*CCL3L1* >4 copies	1.91	1.06–3.41	0.030

Full model shows results from a logistic regression model including all the indicated predictors while final model indicates the results from the stepwise regression using a retention criterion of 0.1; OR, Odds Ratio; CI, Confidence Interval.

The results in Table 1 indicated that none of the *CCR5* haplotypes had a significant association with the risk of KD. In previous studies, we found that the copy number of *CCL3L1* modified the SLE-, Kawasaki disease-, and HIV-1-disease-influencing effects of *CCR5* haplotypes ([15], [37] and data not shown). Thus, one possibility was that the association of *CCR5* haplotypes with KD susceptibility is present only when it is present in the context of a specific *CCL3L1* copy number. To assess this possibility, we conducted the analysis shown in Table 2. We found that *CCR5* haplotypes did not influence KD susceptibility in subjects possessing <4, >4 or 4 copies of *CCL3L1* (Table 2).

**Table 2:** Lack of a modifying influence of the *CCL3L1* gene copy number on the association of five common *CCR5* haplotypes found in the study population with the risk of KD.

*CCR5* haplotype	<4 *CCL3L1* copies		4 *CCL3L1* copies		>4 *CCL3L1* copies		M-H Test	
	OR	95% CI	OR	95% CI	OR	95% CI	χ^2^	P
HHA	0.41	0.01–4.34	5.06	0.34–72.7	1.38	0.35–4.87	2.75	0.2531
HHC	1.22	0.54–2.81	0.93	0.30–3.10	0.65	0.32–1.33	1.54	0.4627
HHE	0.86	0.39–1.86	1.24	0.40–3.78	0.98	0.50–1.89	0.35	0.8398
HHF*1	---	---	0.00	0.00–6.08	0.48	0.01–4.42	3.08	0.2139
HHF*2	0.92	0.42–1.98	0.66	0.21–2.02	0.98	0.50–1.91	0.43	0.8064

The last column shows the results of Mantel-Haenszel test for homogeneity of results across *CCL3L1* copy number.

In our cohort, of the 25 subjects who were resistant to IVIG, 18 (72%) developed CAL. By contrast, of the 68 who responded to IVIG treatment, only 5 (7.3%) developed CAL. This association between IVIG resistance and CAL development was highly significant (Fisher's exact p = 1.4×10^−9^). Evaluation of the association for the outcome of CAL revealed that possession of the *CCR2*-64I-bearing *CCR5* HHF*2 haplotype was associated with a reduced risk of developing CAL which trended towards statistical significance (OR = 0.44, 95% CI = 0.18–1.07, p = 0.071). However, we did not observe a significant association between *CCL3L1* copy number and the risk of developing CAL.

We next determined whether *CCR5* haplotypes and *CCL3L1* copy number associated with IVIG responses. In the full model (Table 3), possession of *CCR5* HHF*2 haplotype was associated with beneficial IVIG responses (OR = 0.21, 95% CI  = 0.54–0.83, p = 0.026). We also found that possession of <4 *CCL3L1* copies was significantly associated with an increased risk of IVIG resistance (OR = 10.93, 95% CI = 1.17–101.99, p = 0.036). Although possession of >4 *CCL3L1* copies was also associated with an increased risk of IVIG resistance (OR = 5.12, 95% CI  = 0.57–46.34, p = 0.146) (Table 3, Full Model), this did not achieve statistical significance. In the final model (Table 3), possession of <4 *CCL3L1* copies remained associated with an increased risk of IVIG resistance (OR = 2.56, 95% CI  = 0.96–6.87, p = 0.061) while possession of *CCR5* HHF*2 haplotype was associated with a salutary IVIG response (OR = 0.34, 95% CI  = 0.12–0.95, p = 0.040). Departure from the population average of 4 copies (i. e, < or >4 copies) was associated with a higher risk of IVIG resistance (OR = 6.26, 95% CI 0.76–51.9, p = 0.089).

**Table 3:** Association of *CCR5* haplotypes and *CCL3L1* copy number with IVIG response.

*CCR5 haplotype/CCL3L1 copy number*	OR	95% CI	P value
Full Model			
*CCR5* HHA	0.83	0.12–5.79	0.851
*CCR5* HHC	0.62	0.15–2.52	0.499
*CCR5* HHE	0.45	0.13–1.51	0.194
*CCR5* HHF*2	0.21	0.54–0.83	0.026
*CCL3L1* <4copies	10.93	1.17–101.99	0.036
*CCL3L1* >4 copies	5.12	0.57–46.34	0.146
Final Model (Probability Criterion of 0.1)			
*CCL3L1* <4copies	2.56	0.96–6.87	0.061
*CCR5* HHF*2	0.34	0.12–0.95	0.040

Full model shows results from a logistic regression model including all the indicated predictors while final model indicates the results from the stepwise regression using a retention criterion of 0.1; OR – Odds Ratio; CI – Confidence Interval.

Because we observed that the *CCR5* HHF*2 haplotype was associated with a reduced risk of IVIG resistance as well as development of CAL, we next examined whether these associations were due to homozygosity and/or heterozygosity of the HHF*2 haplotype. We observed that heterozygosity but not homozygosity for HHF*2 was associated with a reduced risk of both CAL (OR = 0.37, 95% CI 0.14–0.97, p = 0.042) and IVIG resistance (OR = 0.39, 95% CI 0.14–1.11, p = 0.078).

## Discussion

Our results suggest that in Japanese children, copy number variation of the segmental duplication bearing *CCL3L1* associates with susceptibility to KD and IVIG response whereas the *CCR2-64I-*containing CCR5-HHF*2 haplotype is associated with a reduced risk of both CAL development and IVIG resistance. Our finding that deviation from the average *CCL3L1* copy number (i.e., < or >4 copies) found in the Japanese population is associated with increased risk of KD is noteworthy because we have previously found that deviation from median copy number of *CCL3L1* is also associated with an increased risk of systemic lupus erythematosus (SLE) [37] – a disease with broad immunological underpinnings – in three separate cohorts (TX, USA; Ohio, USA; and Medellin, Colombia). The notion that haploinsufficiency and higher gene dosages of immune response genes may influence susceptibility to immune-mediated diseases is also highlighted by our recent observation that both low and high copy numbers of the gene encoding *FCGR3B* was associated with increased susceptibility to SLE and primary Sjogren's syndrome [38]. Together these observations underscore the concept that departure of the gene copy number from a homeostatic copy number, i.e., higher or lower than the average found in the population, may be an important determinant of susceptibility to diseases with a strong immunologic component.

The precise mechanistic basis by which deviation from the average copy number of the *CCL3L1*-containing segmental duplication in our study population was associated with increased KD susceptibility as well as an increased risk of IVIG failure is unknown. As noted, CCL3L1 is the most potent CCR5 ligand and CCR5 ligands are associated with pro-inflammatory effects [39]. Additionally, a copy number of the *CCL3L1*-containing segmental duplication that is higher than the population average is associated with increased leukocyte chemoattraction [29], circulating levels of CCL3 [27] and *CCL3L1* transcript (data not shown). In this light, it is conceivable that subjects bearing higher *CCL3L1*-containing segmental duplications may express higher levels of chemokines following antigenic stimulus that in turn may increase the risk of developing KD and possibly, IVIG resistance. In addition to causing an immunologic blockade of Fc receptor and inducing further antibody production, IVIG therapy is also known to play an important role in down-regulation of the cytokine/chemokine levels [40]. Conceptually then, in persons possessing high *CCL3L1* gene copy numbers the currently used dose of IVIG may be insufficient to induce the desired degree of down-regulation of chemokines leading to IVIG resistance. The latter along with increased CCL3L1 associated inflammation may provide a partial explanation as to why we observed a trend for possession of a high *CCL3L1* copy number and reduced clinical responsiveness to IVIG.

On the other hand, a low *CCL3L1* copy number is associated with reduced CCL3-CCL3L1 chemokine expression levels [29] resulting in reduced inflammatory responses. It has been shown that there is a surge in levels of several cytokines/chemokines during the acute phase of KD [40], [41], [42], [43], [44] and we have observed that the CCL3 surge is a key feature of the acute phase of KD (data not shown). Thus, it is possible that an impaired CCL3-CCL3L1-dependent inflammatory response may partly explain increased risk of KD and reduced clearance of antigen. Consequently, the increased and decreased inflammation associated with a high and low *CCL3L1*-containing segmental duplication, respectively, may explain why all subjects do not respond to a single dose of IVIG and require additional treatments. This hypothesis is supported by the fact that greater than half of IVIG-resistant patients who receive an additional dose of IVIG become afebrile [10]. While appealing, laboratory or clinical data that directly evaluates these hypotheses regarding mechanisms by which a high or low *CCL3L1* gene copy associates with KD and IVIG non-responsiveness are currently lacking and require validation.

The role of *CCR5* polymorphisms in KD susceptibility has been investigated previously. Significant attention has focused on the widely recognized 32-bp deletion (Δ32) mutation present in the coding region of *CCR5* that is found mainly in populations of European ancestry. We reported previously that there was an inverse relationship between the global distribution of Δ32 allele and the incidence of KD [15]. Also, in our large family-based study in US-trios we had observed an asymmetric transmission of the *CCR5*-Δ32 allele across generations [15]. Further, we had found that the KD-influencing effects of the *CCR5*-Δ32-bearing HHG*2 haploype were modified by *CCL3L1* copy number [15]. Breunis et al [26] replicated our observations in a Northern European population and observed that the frequency of the *CCR5-*Δ32 allele was lower in cases (6.5%) compared to controls (10.7%).

The *CCR5-*Δ*32*-bearing HHG*2 haplotype is rarely found in Asian populations. The results of two prior studies in subjects with KD of European ancestry [15], [26] and a separate study of KD patients from Korea [19] suggested that other polymorphisms at the *CCR5* locus also associate with susceptibility to KD. However, in the present study of Japanese subjects, we did not find an association between *CCR5* haplotypes and KD susceptibility. By contrast, we did find an association of *CCR5* haplotypes with KD outcomes and IVIG-resistance.

Early coronary lesions demonstrate marked infiltration of neutrophils [45] whereas at later time points show infiltration predominantly of T-cells and monocytes/macrophages [43]. Members of the chemokine system, including CCR5 and CCL3L1 play an important role in leukocyte trafficking and activation as well as the pathogenesis of coronary artery diseases such as arteriosclerosis, hypertension and myocardial infarction [46]. In our previous study of European-descent KD patients, we found that the Δ32-bearing *CCR5*-HHG*2 haplotype was associated with not only reduced KD susceptibility, but also a lower risk of CAL [15]. In the present study, we observed that the *CCR5* HHF*2 haplotype which bears the CCR2-64I polyrmorphism is associated with a reduced the risk of IVIG-resistance and CAL formation. Whether this association suggests an involvement of CCR2, a receptor critically involved in monocyte trafficking and activation, in KD pathogenesis and therapy responses is unclear because the *CCR2*-64I polymorphism is in linkage disequilibrium with promoter polymorphisms in *CCR5* [32]. Notwithstanding this quandary, it is conceivable that the beneficial associations observed for the CCR2-64I-bearing *CCR5* HHF*2 haplotype with KD-related outcomes may relate either directly or indirectly to inflammation.

Many demographic and laboratory factors such as patient age, white blood cell count, and plasma levels of aspartate amino transferase and C-reactive protein have been identified as risk factors for IVIG resistance [3], [6], [8], [47], [48], [49]. Onouchi et al [22] observed that a functional polymorphism in the *ITPKC* gene was associated with response to IVIG in US KD children. The results of the present study extend the notion that host genetic factors may influence IVIG resistance. IVIG has been shown to be effective across a range of autoimmune, inflammatory and infectious conditions, as well as for post-infectious complications [25]. This suggests that IVIG may have a broad immunomodulatory mechanism of action, beyond merely inhibiting antibody-triggered inflammation. Park-Min et al showed recently that IVIG blocks cellular activation by interferon-γ (IFNγ) [50], a proinflammatory cytokine that plays a key role in cellular immune responses and Th1-type-driven inflammatory/infectious diseases [51], [52]. In this respect it is notable that CCR5 is expressed on Th1 cells [53], and thus it is conceivable that polymorphisms in this gene and its ligands by influencing Th1 pathways may influence IVIG responses. Because IVIG is far from an optimized therapeutic, and responses to IVIG vary considerably among patients, future studies are warranted to identify the broader range of host genetic factors that underlie the observed inter-subject differences in IVIG responses.

Although a limitation of our study is the small sample size, our results are concordant with previous data suggesting a role for variations in *CCL3L1* and *CCR5* in KD [15], [19], [26]. Recent data suggest that the Japanese population may not be as homogeneous [54] as once thought and the possibility of population stratification exists since our controls and cases came from different regions of Japan. However, despite this limitation it is noteworthy that similar to the *CCR5*-Δ32 polymorphism which has been intensively scrutinized in persons of European descent, the *CCR2*-64I polymorphism has also been associated with variable susceptibility to multiple diseases as well. Interestingly, while the *CCR5*-Δ32 polymorphism is rare among persons of Asian descent, the *CCR2*-64I-containing HHF*2 haplotype is very common, and has been associated with salutary effects (reduced risk) among persons of Japanese descent for several diseases with immunologic underpinnings including multiple sclerosis [55], sarcoidosis [56], and HIV [57]. Also, it is well-known that CAL is associated with IVIG resistance [10], [47] and concordantly, we found that IVIG-resistant subjects had a higher proportion of CAL and that the *CCR2*-64I-containing HHF*2 haplotype was associated with beneficial effects for both of these outcomes. Another limitation of our study is that we had to consider CAL outcomes as a dichotomous variable as the clinical centers in Japan did not uniformly use Z scores to characterize the dimension of the lumen of the coronary arteries [58]. Analyzing these data with arterial internal diameter normalized for body surface area as a continuous variable may have yielded more robust results.

Notwithstanding these limitations, our findings underscore that genetic determinants influence not only inter-individual differences in KD susceptibility but also inter-subject variation in cardiac complications and treatment response. In conjunction with previous studies that have focused on the relationship between variations in CCR5 and its ligands with KD [15], [19], [26], the current findings showing a commonality of the genetic associations across three different KD-related phenotypes (KD susceptibility, CAL development and IVIG resistance) together suggest that CCR5 and its ligands may play an important role in the pathogenesis of different facets of KD. Of broad importance, our findings also suggest that host variations may influence responses to immune modulators such as IVIG.
